# Convergence of eicosanoid and integrin biology: 12-lipoxygenase seeks a partner

**DOI:** 10.1186/s12943-015-0382-5

**Published:** 2015-06-03

**Authors:** Keqin Tang, Yinlong Cai, Sangeeta Joshi, Elizabeth Tovar, Stephanie C. Tucker, Krishna Rao Maddipati, John D. Crissman, William T. Repaskey, Kenneth V. Honn

**Affiliations:** Department of Radiation Oncology, John D. Dingell VA Medical Center, 48201 Detroit, MI USA; Department of Pathology, Bioactive Lipids Research Program, Wayne State University School of Medicine, Karmanos Cancer Institute, 431 Chemistry Building, 48202 Detroit, MI USA; Program in Cancer Biology, Wayne State University School of Medicine, 48202 Detroit, MI USA; Department of Internal Medicine, University of Michigan, 48109 Ann Arbor, MI USA; Present address: Roswell Park Cancer Institute, 14263 Buffalo, New York USA; Present address: Van Andel Institute, 49503 Grand Rapids, MI USA

**Keywords:** 12-lipoxygenase, α6β4 integrin, Eicosanoid, Migration, Apoptosis

## Abstract

**Background:**

Integrins and enzymes of the eicosanoid pathway are both well-established contributors to cancer. However, this is the first report of the interdependence of the two signaling systems. In a screen for proteins that interacted with, and thereby potentially regulated, the human platelet-type 12-lipoxygenase (12-LOX, *ALOX12*), we identified the integrin β4 (*ITGB4*).

**Methods:**

Using a cultured mammalian cell model, we have demonstrated that ITGB4 stimulation leads to recruitment of 12-LOX from the cytosol to the membrane where it physically interacts with the integrin to become enzymatically active to produce 12(S)-HETE, a known bioactive lipid metabolite that regulates numerous cancer phenotypes.

**Results:**

The net effect of the interaction was the prevention of cell death in response to starvation. Additionally, regulation of β4-mediated, EGF-stimulated invasion was shown to be dependent on 12-LOX, and downstream Erk signaling in response to ITGB4 activation also required 12-LOX.

**Conclusions:**

This is the first report of an enzyme of the eicosanoid pathway being recruited to and regulated by activated β4 integrin. Integrin β4 has recently been shown to induce expansion of prostate tumor progenitors and there is a strong correlation between stage/grade of prostate cancer and 12-LOX expression. The 12-LOX enzymatic product, 12(S)-HETE, regulates angiogenesis and cell migration in many cancer types. Therefore, disruption of integrin β4-12LOX interaction could reduce the pro-inflammatory oncogenic activity of 12-LOX. This report on the consequences of 12-LOX and ITGB4 interaction sets a precedent for the linkage of integrin and eicosanoid biology through direct protein-protein association.

**Electronic supplementary material:**

The online version of this article (doi:10.1186/s12943-015-0382-5) contains supplementary material, which is available to authorized users.

## Background

The human β4 integrin subunit was identified as a 12-LOX-interacting protein, and thus a potential 12-LOX regulator [1]. Integrins are multi-domain glycoproteins that promote cellular adhesion, and coordinate growth and differentiation signals. The β4 integrin subunit is part of a cell surface receptor (α6β4) for laminin (LN), an extracellular matrix component. Ligation of this surface receptor by LN or by an activating antibody (3E1), triggers signaling pathways involved in cell proliferation, differentiation, apoptosis, adhesion, invasion and metastasis [2]. β4 impacts angiogenesis [3], anchorage-independent growth [4], cell survival [5], cellular invasion [6], and tumor progression [7]. Integrin β4 is associated with increased cancer aggressiveness [8], which is likely due to its ability to cooperate with other receptors [9–12].

12-Lipoxygenase is associated with many of the same β4-mediated phenotypes [13], and also promotes tumor cell survival. Lipoxygenases (LOX) are a family of non-heme iron-containing dioxygenases that stereo specifically insert molecular oxygen into 1,4-cis, cis-pentadiene-containing polyunsaturated fatty acids to ultimately produce bioactive lipids such as leukotrienes, lipoxins, jasmonates and 12-hydroxyeicosatetraenoic acid [12(S)-HETE] that regulate numerous biological and pathological processes [13, 14]. The platelet-type 12-LOX (P-12-LOX) is one of three mammalian 12-LOX isoforms (classified as platelet-, leukocyte-, or epidermal-type) that differ in tissue distribution, substrate preference, and metabolite profile, and is notably elevated in a variety of human tumors where it is anti-apoptotic [15, 16]. In a clinical study, 38 % of the prostate cancer patients studied (n = 132) exhibited elevated levels of P-12-LOX in cancer tissues, which correlated positively with tumor stage, grade and positivity for prostate cancer cells in the surgical margins [17].

12-LOX metabolizes arachidonic acid (AA) exclusively to 12(S)-HETE [18]. This metabolite is intimately linked to tumor progression and metastasis as well as to other pathological conditions, such as psoriasis, atherosclerosis and arthritis [13, 19–21]. 12(S)-HETE modulates integrins (e.g., αvβ3), regulates secretion of proteinases, enhances tumor cell motility and invasion, and induces angiogenesis [13, 22, 23], which represent traits that are also regulated by ITGB4. 12-LOX enzymatic activity is also regulated by subcellular compartmentalization, and there is precedent for the enzymatic activity of other lipoxygenases, namely 5-LOX and 15-LOX, being compartment-dependent [24, 25]. In the present study we utilized a cell culture model to characterize both the physical interactions between 12-LOX and the β4 integrin subunit and the functional outcomes of these interactions. This is the first report of an integrin regulating an enzyme of an eicosanoid biosynthetic pathway, and suggests a new paradigm for both integrin and eicosanoid biology.

## Results

### The cytoplasmic domain of β4 interacts with 12-LOX in tumor cells

**Fig. 1:**
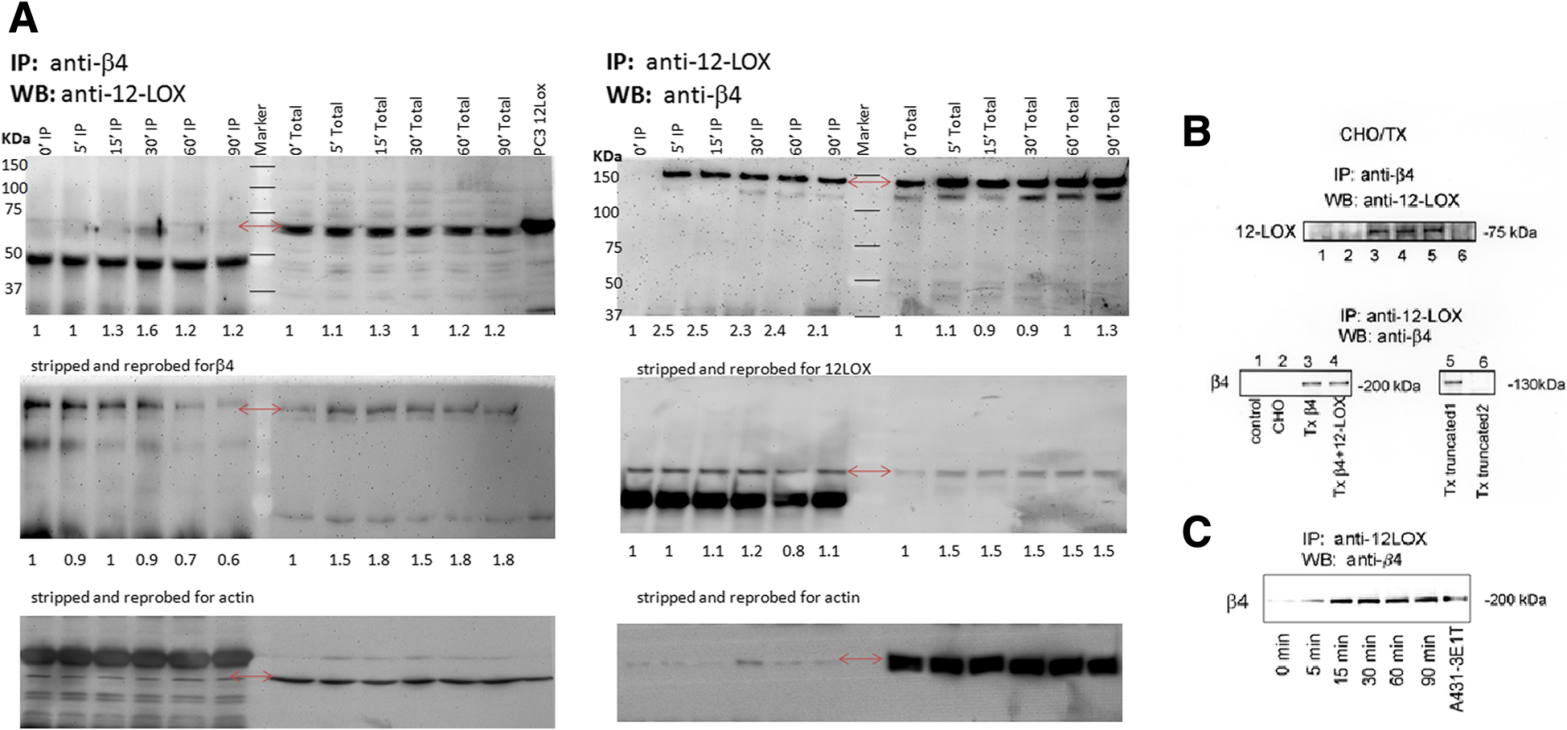
P-12-lipoxygenase interacts with integrin β4 subunit *in vitro*. (**a**) A431 cells were stimulated with mAb β4 3E1 and harvested at timed intervals from 5–90 min. 12-LOX and the β4 subunit co-immunoprecipitated from untransfected A431 cells (A431), [and A431 cells over expressing 12-LOX (Tx-A431)-Additional file 1]. (**b**) CHO cells were transfected with different β4 constructs either alone or in combination with 12-LOX: (1) *control*: vector alone; (2) *CHO:* nontransfected cells; (3) *Txβ4:* full-length β4; (4) *Txβ4 + 12-LOX:* cotransfected with full-length β4 and 12-LOX; (5) *Txtruncated1:* pCMV-β4 Δ70-660 (c-myc tagged head-less) *+12-LOX*; (6) *Txtruncated2:* pCMV-β4 Δ854-1752 (tail-less) *+12-LOX*. 12-LOX and the β4 subunit coimmunoprecipitated from three transfectants: Txβ4, Txβ4 + 12-LOX, and Txtruncated1 (headless). Positions of 12-LOX and β4 are indicated. The experiment was repeated three times. For each experiment, mouse or rabbit IgG (control 1) and Sepharose 4B-conjugated protein G beads alone (control 2) were used as negative controls. (**C**) 12-LOX and the β4 subunit co-immunoprecipitated from A431 cells after growth on laminin at the same timed interval as with mAb 3E1 stimulation. Whole cell lysate of A431 stimulated with mAB 3E1 was loaded as a control (A431-3E1T). β4 subunit was detected with mAb 450-11A

The A431 human epidermoid carcinoma cell line has been widely used to study 12-LOX, as it expresses enzymatically active 12-LOX protein, but not the leukocyte-type isoform [24]. Previously we reported that 12-LOX interacts with the C-terminal cytoplasmic domain of the integrin β4 subunit in a yeast-two hybrid screen of an A431 library [1]; an interaction that was validated in the parental A431 cells and 12-LOX over-expressing transfectants. We performed a co-immunoprecipitation assay to examine the endogenous interaction of 12-LOX with β4. Cells were stimulated with an antibody to the extracellular domain of β4 subunit (3E1), and β4-associated proteins were subsequently immunoprecipitated from extracts using antibodies that either recognized the extracellular domain (3E1, 439-9B) or the cytoplasmic domain of β4 (450-11A), and these were probed for the presence of 12-LOX. In reciprocal experiments, following stimulation with 3E1, samples were first immunoprecipitated with anti-12-LOX antibody, and then probed for β4 association. In A431 cells and A431 12-LOX transfectants (Additional file 1), we detected 12-LOX immunoprecipitated with a mAb to β4 (Fig. 1*A*, *left panels*). Conversely β4 was immunoprecipitated with an antibody to 12-LOX (Fig. 1*A*, *right panels*). The association of 12-LOX with β4 was time-dependent beginning at 5 min post stimulation. These data suggest that the interaction between 12-LOX and β4 originally discovered in the yeast 2-hybrid model system also occurs in a cultured human tumor cell model.

We tested this interaction in CHO cells that express α6β1 and low levels of 12-LOX, but not β4 [26]. After confirming ectopic co-expression of β4, or truncated β4, with 12-LOX from cDNA constructs in CHO cell transfectants, we found that 12-LOX co-immunoprecipitated with β4, and vice versa (Fig. 1*B*, *upper and lower panel, respectively. lane 3-CHO transfectants producing full-length, wild-type β4 alone, or lane 4-in combination with 12-LOX)*. Full-length β4 was recognized by antibodies to both its extracellular (3E1 mAb) and cytoplasmic (450-11A mAb) domains. 12-LOX also co-immunoprecipitated with truncated, head-less β4 (Fig. 1*B*, *lane 5*, *upper and lower panel*), but not with truncated, tail-less integrin β4 (Fig. 1*B*, *lane 6*, *upper and lower panel*). While truncated, tail-less β4 (95 kDa) complexes with α6 [26], in transfectants with tail-less β4, 12-LOX did not co-immunoprecipitate with the mAb to the cytoplasmic domain of the β4 (450-11A) or with either mAb to the extracellular domain of β4, namely 3E1 or 439-9B (Fig. 1*B lane 6*, *upper and lower panel*). In contrast, in transfectants expressing truncated, headless β4, 12-LOX co-immunoprecipitated with the 130-kDa truncated β4, which was detectable with the 450-11A mAb to the cytoplasmic tail (Fig. 1*B*, *lane 5, lower panel*), but not with mAbs 3E1 or 439-9B. These findings suggest that 12-LOX associates with the cytoplasmic domain of β4 and that it interacts with β4 when the two proteins are ectopically expressed in CHO cells. The differential immunoprecipitation of 12-LOX with the panel of β4 constructs suggests that the cytoplasmic domain of β4 is crucial for its interaction with 12-LOX, thus confirming our earlier yeast two-hybrid results and those observed with endogenous β4 in A431 cells.

Integrin β4 was detected in Western blots at the expected molecular mass of 200 kDa. Interestingly, two minor bands of 135 and 170 kDa from immunoprecipitates of 12-LOX from both A431 cells and CHO transfectants were also detected with β4 antibodies when the Ca^2+^ concentration in the lysis buffer was high, which is in agreement with a previous report [26] that suggested the cytoplasmic domain of β4 is susceptible to a calcium-dependent protease present in cellular extracts. Sequence analysis reveals two calpain cleavage (PEST) sites that could result in the minor bands we detected.

Finally, to verify the report that the mAb 3E1 functionally stimulates β4 as well as laminin, the natural ligand, immunoprecipitation was done with 12-LOX antibody on laminin-treated cells with similar results Fig. 1*C*.

### β4 ligation-induced translocation of 12-LOX in A431 cells

**Fig. 2:**
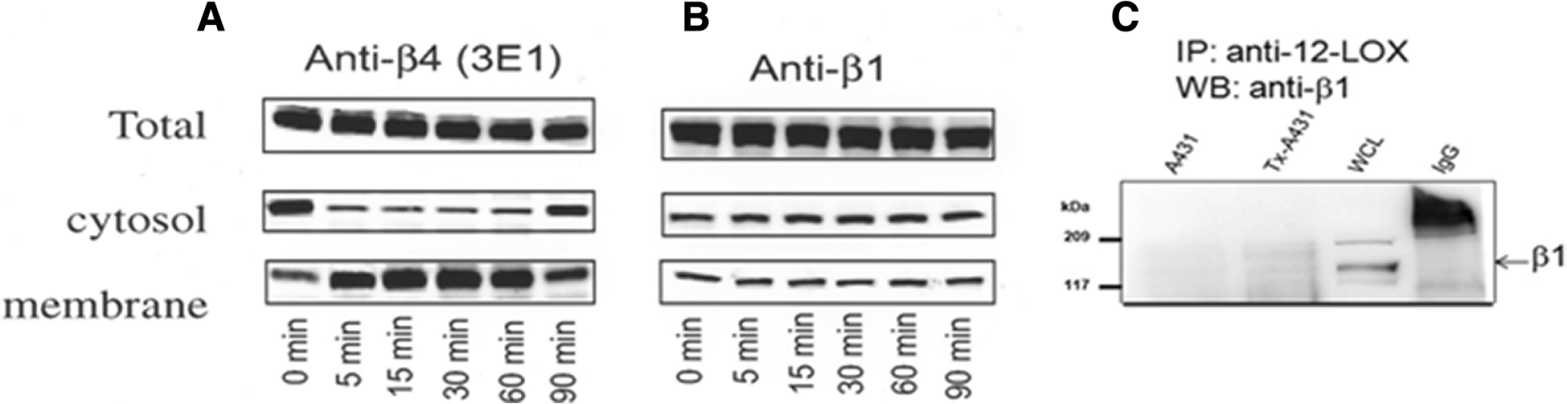
β4 ligation-induced translocation of 12-LOX in A431 cells. A431 cells stimulated with 3E1 mAb for 5, 15, 30, 60 and 90 min. (**a**) Membrane translocation of 12-LOX from cytosol to membrane. (**b**) The effect of β4 stimulation on 12-LOX translocation is specific to β4. (**c**) β1 integrin is detectable in whole cell lysates (WCL), but does not interact with 12-LOX on β4 stimulation

Previous studies demonstrated that EGF, Ca^2+^ and the phorbol ester TPA (12-O-tetradecanoylphorbol-13-acetate) increased 12-LOX activity by inducing its translocation from cytosol to membrane [24, 25, 27]. We examined whether β4 interaction altered translocation of 12-LOX in A431 cells where β4 was stimulated with 3E1 mAb for 5, 15, 30, 60 and 90 min. Membrane translocation of 12-LOX from cytosol was observed within 5 min, peaked by 15 min, and was sustained for 60 min (Fig. 2*A*). Thereafter the response was down-regulated. The effect of β4 stimulation on 12-LOX translocation was specific as β1 stimulation with activating antibody (Fig. 2*B*) failed to induce any significant increase in membrane-associated 12-LOX. Furthermore, while detectable in whole cell lysates, β1 did not interact with 12-LOX on β4 stimulation (Fig. 2*C*). In all experiments, total 12-LOX protein level was unaltered after treatment with any of the antibodies used in this study (Fig. 2, *upper panels-Total*). The biochemistry was validated with confocal immunofluorescence data (Additional file 2, Additional file 3, Additional file 4, Additional file 5, Additional file 6 and Additional file 7). Collectively these results demonstrate for the first time that β4 ligation induces the translocation of 12-LOX from cytosol to membrane where the two proteins interact.

### The interaction of β4 with 12-LOX upregulates 12-LOX enzymatic activity

**Fig. 3:**
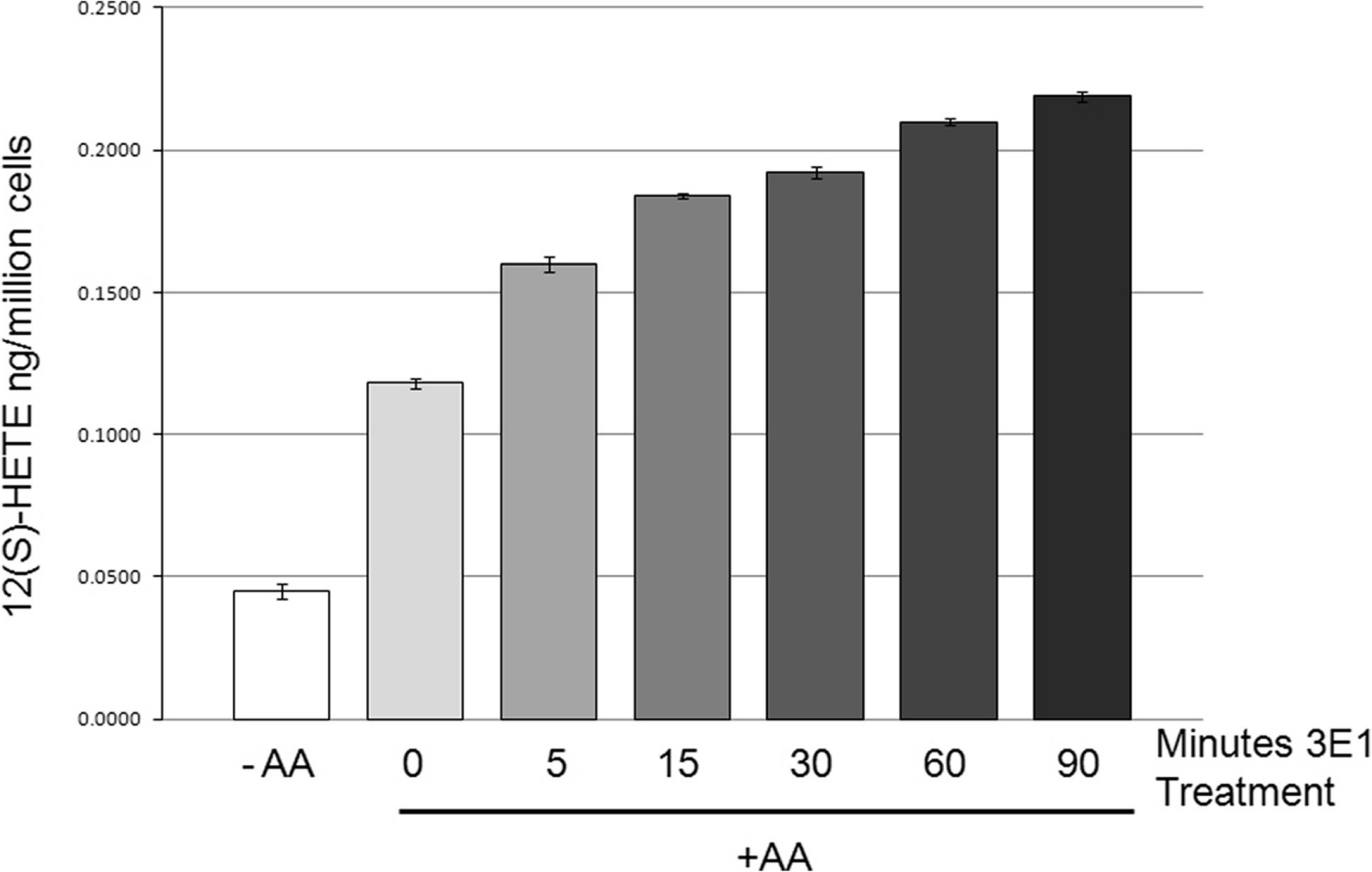
LC-MS analysis of 12-LOX activity. Following the stimulation with mAb β4 over a time course, each membrane fraction (100,000 × g pellet) was harvested and incubated in DMEM at 37 °C with 10 μM [^14^C]-AA for 15 min. Cell lipids were extracted and analyzed as described in Materials and Methods. The data were analyzed by LC-MS in triplicate and error bars represent SEM

To study whether 12-LOX activity is altered following translocation to β4, its enzymatic activity was determined by LC/MS (Fig. 3) or RP-HPLC (Additional file 8) analyses for its sole arachidonate metabolite, 12(S)-HETE. LC/MS measurements of total cellular and secreted 12(S)-HETE were made. As with the isolated membrane fractions, there is an accumulation of 12(S)-HETE metabolite with 3E1 stimulation (Fig. 3). Cytosolic and membrane protein fractions were isolated from A431 cells following 5, 15, 30, 60 and 90 min treatment with 3Ε1 or control IgG. Subsequently each fraction was incubated with 10 μM [^14^C] AA in DMEM, followed by lipid extraction as described. In accordance with a previous study [24], RP-HPLC analysis indicated that 12(S)-HETE was the major product formed from exogenous AA in these subcellular fractions, whereas other HETEs including 5-HETE and 15-HETE were not detected under our conditions (Additional file 8*a*). Of the total 12-LOX activity, 75 ± 6 % (mean ± s.d.; n = 3) resided in the membrane fraction (100,000 x g-pellet), 20 ± 9 % in the 10,000 × g-pellet, and only 5 ± 1.2 % in the cytosolic fraction. β4 ligation increased membrane-bound 12-LOX and enhanced its activity in a time-dependent manner, starting at 5 min and peaking at 60 min when 12(S)-HETE production was 4–5 fold higher than control (mouse IgG) (Additional file 8*b*). In agreement with the membrane translocation experiment, 12(S)-HETE production also declined 60 min post β4 mAb stimulation. Therefore, recruitment of 12-LOX to the β4 subunit appears to stimulate enzymatic activity and provides rationale for our earlier observations that membrane-associated 12-LOX was the dominant enzymatically active form in A431 tumor cells [24].

### Activation of 12-LOX by β4 stimulation blocks A431 cells from apoptosis induced by 12-LOX inhibitor

**Fig. 4:**
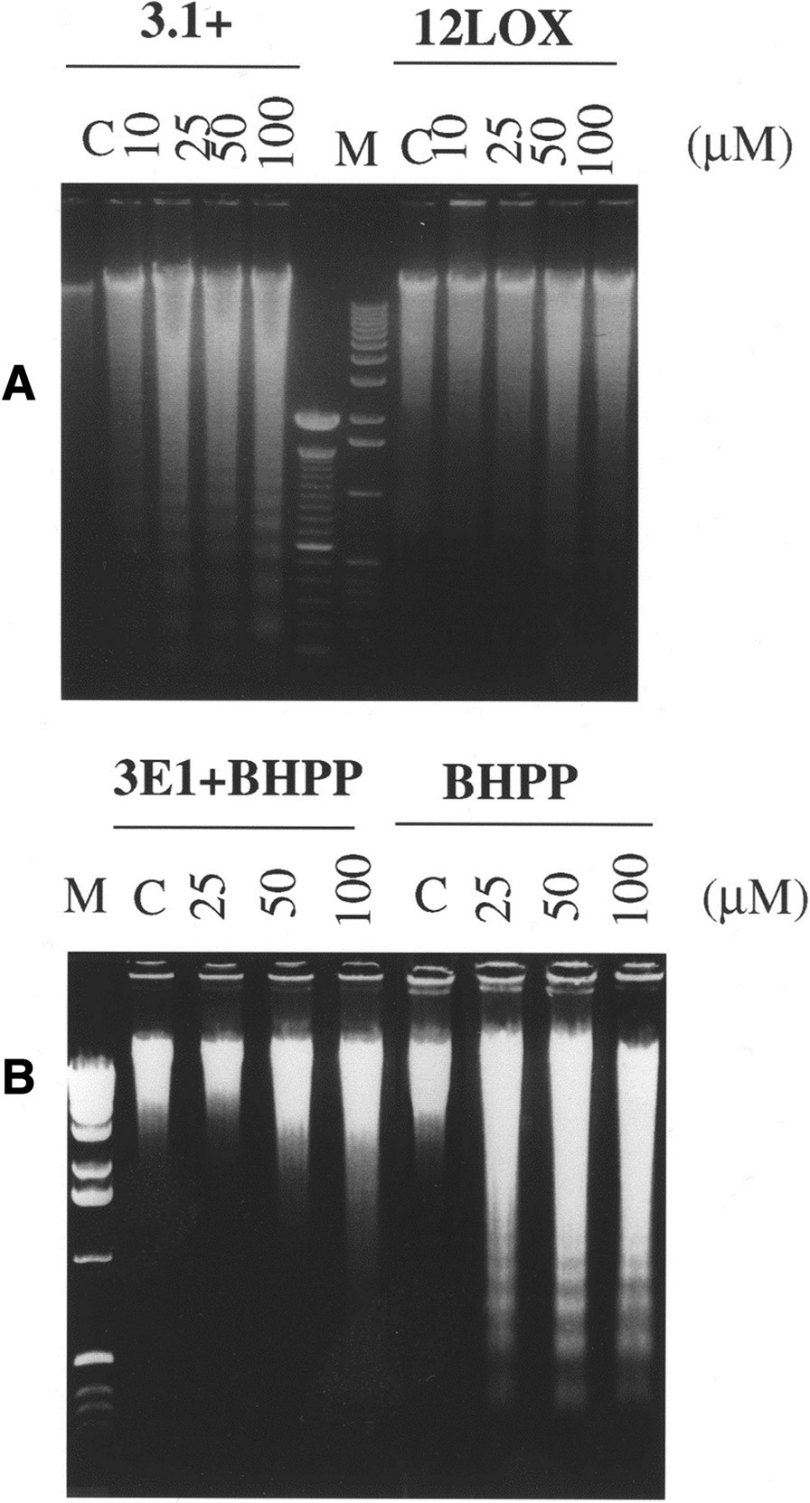
mAb β4 treatment effect on BMD122-induced apoptosis in A431 cells by DNA laddering assay. (**a**) Comparison of A431 12-LOX transfectants with empty vector control (3.1+). Cells were treated with BMD122 (formerly BHPP) at the concentration indicated for 24 h, low molecular weight DNA was extracted, run on a 1.5 % agarose gel and visualized with ethidium bromide. The middle lanes are DNA markers for comparison. *3.1+*: empty vector controls; *12LOX*: A431 cells transfected with full-length 12-LOX. (**b**) A431 cells were pretreated with mAb β4 3E1 (5 μg/ml) before incubation in DMEM in the presence of BMD122, see details in Materials and Methods. Aliquots of DNA extracts were subjected to 1.5 % agarose gel and visualized with ethidium bromide. *C*: Ethanol as vehicle control. *M*: DNA marker, left lane

Lipoxygenase metabolites may act as survival factors in a variety of tumor cells, as has been suggested [28]. As the interaction of 12-LOX with the cytoplasmic domain of β4 led to elevated levels of 12(S)-HETE, we explored whether this interaction may contribute to cancer cell survival. A DNA laddering assay revealed that pharmacological inhibition of 12-LOX with BMD122 induced apoptosis in A431 cells in a dose-dependent manner, similar to the effects found in an earlier study with W256 cells [28] (Fig. 4*B*). This suggests that 12-LOX is anti-apoptotic in A431 cells. Compared to parental A431 cells, or vector controls, only 12-LOX transfectants were more resistant to apoptosis induced by BMD122 as shown by the density of the DNA ladder (Fig. 4*A*). Pre-incubation of A431 cells with mAb β4 3E1 for two hours prior to the treatment with BMD122 (Fig. 4*B*), completely prevented cells from undergoing BMD122-triggered apoptosis at low BMD122 concentrations and significantly protected cells exposed to high dose BMD122 (Fig. 4*B*). Both ELISA detection of cytoplasmic nucleosomes [29] and Trypan blue-exclusion ([30] and references therein) were employed as an alternate measure of cell death (Additional file 9 and Additional file 10).

### 12-LOX activation by β4 mediates EGF-stimulated migration of A431 cells

**Fig. 5:**
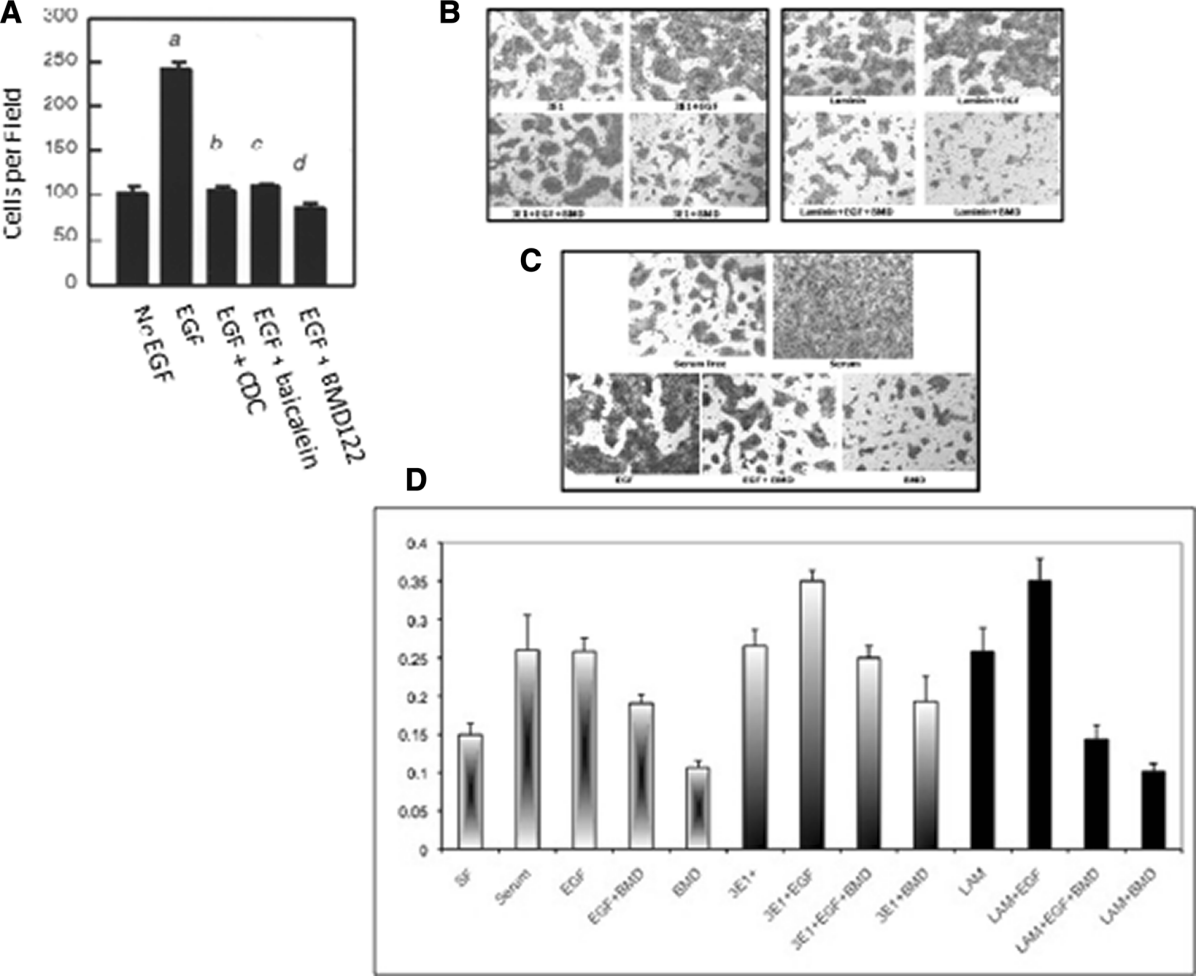
The α6β4 integrin and 12-LOX activity function in EGF-induced chemotaxis. (**a**) Migration assay: A431 cells were pretreated with 3E1 antibody for 20 min, then plated on laminin-coated invasion plates either with or without EGF (1 ng/ml) for 3 h in the presence or absence of pharmacological inhibitors as described in Methods. Data represent the mean number of transmigrated cells/microscopic field (± SE). The experiments were repeated three times in triplicate. *a*, P < 0.01 when compared to no-EGF treated group; *b, c, d*, P < 0.01 when compared to EGF treated control group. (**b**) Alternate migration assay: Comparison of chemotaxis of A431 cells toward EGF when stimulated with either mAb 3E1 or laminin in the presence or absence of the 12-LOX specific inhibitor BMD122. (10× magnification) (**c**) Controls for (**b**). (**d**) Absorbance measurements of dye retained by transmigrated cells

EGF enhances α6β4-dependent cell migration of A431 cells on laminin [31]. As 12-LOX interacts physically and functionally with β4 in A431 cells, we tested whether 12-LOX modulates integrin-dependent migration on laminin or Matrigel. We demonstrated that EGF induced A431 cells, preincubated with 3E1, to migrate on laminin by 2–2.5 fold, and inhibition of 12-LOX by pretreatment of cells with specific inhibitors (i.e., CDC, baicalein, or BMD122) reduced A431 cell migration to the level observed in the absence of EGF stimulation (Fig. 5*A*). Laminin and 3E1 antibody induced migration through Matrigel equally well (Fig. 5*B-D*). However, the inhibitory effect of BMD122 on migration was greater in laminin-treated cells (Fig. 5*B*). Our data suggest that α6β4-dependent cell migration on laminin in response to EGF is also regulated by 12-LOX.

**Fig. 6:**
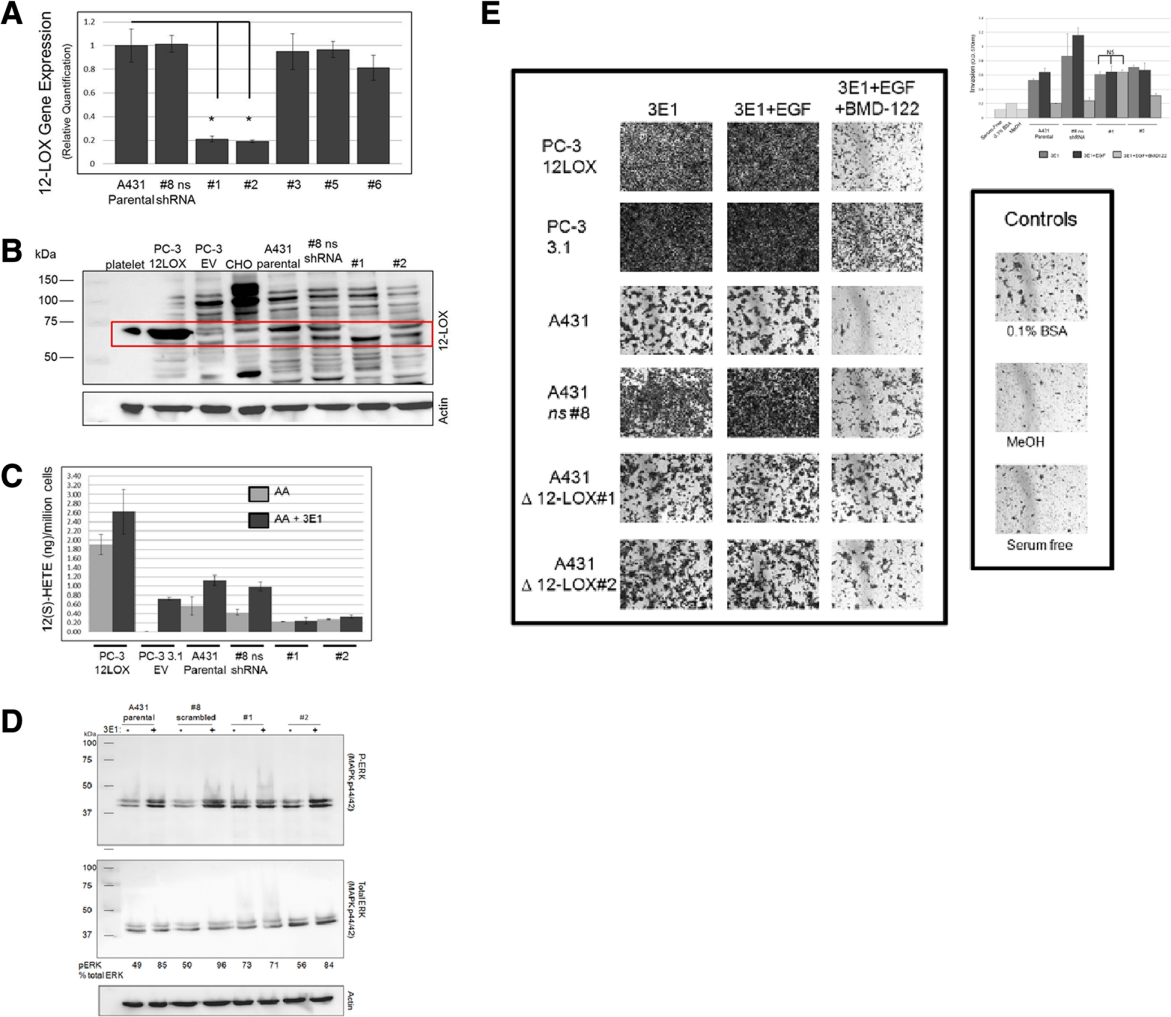
12-LOX knockdown inhibits β4-mediated 12(S)-HETE production, downstream ERK activation, and cellular invasion. (**a**-**c**) Validation of 12-LOX KD in A431 cells. (**a**) 12-LOX mRNA levels measured by RT-PCR in A431 parental, ns (non-silencing) shRNA control cells, and 12-LOX KD cell lines. *p < 0.001 (**b**) Western blot analysis of 12-LOX protein levels in 12-LOX KD clones, ns shRNA control, parental A431, CHO (negative control for 12-LOX expression; polyclonal platelet-type 12-LOX antibody appears to be recognizing another 12-LOX isoform in CHO cells), PC-3 12-LOX overexpressors along (positive control for 12-LOX), 3.1 empty vector control cells, and platelet lysate (positive control for 12-LOX expression). (**c**) No increase in 12(S)-HETE levels were seen with 3E1 stimulation in #1 and #2 12-LOX KD clones. 12-LOX activity was measured by 12(S)-HETE production using LC-MS after a 6 h incubation with 3E1 and AA. (**d**) #1 12-LOX KD cells do not respond to 3E1 stimulation with an increase in phosphorylated ERK levels. Western blot evaluation of phosphorylated ERK with 30 min 3E1 stimulation. Densitometry analysis represents the ratio of phosphorylated ERK to total ERK. (**e**) #1 12-LOX KD cell invasion is not affected by BMD122 enzymatic inhibition of 12-LOX. Cells were pre-treated with BMD122, then stimulated with 3E1 or EGF and allowed to invade through a Boyden Chamber insert coated with Matrigel for 24 h. Images taken at 10 x. Invaded cells were stained with crystal violet, the dye content dissolved in 10 % acetic acid, and the absorbance measured at OD_570nm_. Columns represent the invasion reported as the mean of three samples +/− SE

To confirm the role of 12-LOX in β4-regulated 12(S)-HETE production and EGF-stimulated migration, we transfected A431 cells with six different shRNA constructs, each targeted to a unique region of the 12-LOX gene, and screened for 12-LOX knockdown after puromycin selection. Both 12-LOX gene and protein expression were assayed to validate the knockdown (Fig. 6*A, B*). Compared to the parental and non-silencing (*ns*) shRNA control cells, the #1 and #2 clones showed decreased 12-LOX mRNA expression, as measured by RT-PCR (Fig. 6*A*). None of the cells transfected with construct #4 survived selection, and so these were not included in the analysis. At the protein level, clone #1 appeared to lack 12-LOX compared to the parental and *ns* shRNA control cells (Fig. 6*B*). We also analyzed 12(S)-HETE production with 3E1 stimulation, which we demonstrated activates 12-LOX enzymatic activity (Additional file 8*a*). PC-3 prostate cancer cells stably expressing 12-LOX were used as a positive control for 12(S)-HETE production (Fig. 6*C*). In both the parental A431 and *ns* shRNA control cells, 3E1 stimulation resulted in an increase of 12(S)-HETE production compared to AA treatment alone. This response was not seen in the #1 or #2 clones, indicating that 12-LOX interaction with activated β4 stimulates its enzymatic activity. Downstream of β4 stimulation and subsequent 12-LOX recruitment / enzymatic activation, 12(S)-HETE acts back on its receptor, 12HETER1, so as to activate MAPK signaling [32, 33, 34]. As seen in Fig. 6*D*, parental and *ns* shRNA control cells respond to 3E1 with an increase in ERK phosphorylation. Basal ERK activation was higher in clone #1, which did not increase in response to 3E1. This may be the result of compensatory survival signaling in response to the loss of 12-LOX and its associated pro-survival signals.

Next, we utilized the 12-LOX KD cells to confirm the role of 12-LOX in integrin-mediated, EGF-stimulated cell invasion (Fig. 6*E*). Consistent with previous results, β4 stimulation and EGF increased invasion of the parental and *ns* shRNA control A431 cells and prostate PC3-12LOX transfectants, while BMD122 dramatically reduced invasion. The invasion of the *ns* shRNA cells was increased in all conditions compared to the parental control and could be due to non-specific targeting effects of the scrambled shRNA. EGF stimulation lead to marginal, if any, increased invasion in the #1 and #2 12-LOX KD cell lines. This suggests that 12-LOX promotes EGF-stimulated invasion. Similar to the results seen in the parental and *ns* shRNA cells, BMD122 reduced cell invasion in the #2 12-LOX KD that had residual 12-LOX protein, whereas it had no effect on the #1 12-LOX KD cells. Therefore, despite β4 stimulation, EGF did not effectively stimulate invasion in the absence of 12-LOX.

## Discussion

The platelet-type, metabolically active, 12-LOX is upregulated in a variety of tumor cell types such as Lewis lung and rat Walker carcinoma cells. Furthermore, overexpression of 12-LOX in prostate or breast cancer cells stimulates growth in tumor xenograft models, and tumor angiogenesis [23, 35], where 12-LOX overexpression regulates HIF1α [36]. The sole metabolic product of AA metabolism by 12-LOX, 12(S)-HETE, modulates several traits related to the metastatic potential of tumor cells. These include cell motility [37], secretion of lysosomal proteinases cathepsins B and L [38], expression/secretion of MMP9 [22], invasion [22, 34], expression of integrin receptor αΙIbβ3 [39], tumor cell adhesion to endothelium, and spreading on subendothelial matrix [13]. The role of 12(S)-HETE in tumor cell induced platelet activation (TCIPA) is well-appreciated [40, 41], and additional studies have recently identified 12-LOX as a contributing factor to immune-mediated thrombosis [42]. 12(S)-HETE also regulates lung colonization in vivo. This metabolite activates downstream signaling by virtue of the cognate receptor for 12(S)-HETE (GPR31, 12-HETER1) discovered by our group [34]. However, until now there has been little insight into how the activity of 12-LOX enzyme itself is regulated.

Given that 12-LOX membrane translocation is essential for increased activity, and that the integrin β4 subunit was identified as an interacting protein [1], we hypothesized that 12-LOX interaction with the cytoplasmic domain of the β4 subunit may enhance its lipoxygenase activity, particularly as these proteins contribute to similar cancer phenotypes.

The enzymatic activities of 5-LOX and 15-LOX are also increased by membrane translocation. For example, 5-LOX is predominantly cytosolic in resting neutrophils but translocates to the nuclear membrane [43] to associate with FLAP. Likewise, 15-LOX activity reportedly increases in reticulocytes after membrane translocation [44]. Additional studies support that 12-LOX enzymatic activity in rat W256, HEL, and murine B16a melanoma cells is membrane-associated despite the protein being predominantly cytosolic [24, 45].

The integrin β4 is essential for the organization and maintenance of epithelial architecture through formation of hemidesmosomes that link the intermediate filament cytoskeleton to the extracellular matrix. It is a dynamic protein that also has strong connections to tumor-associated phenotypes such as invasion, angiogenesis, and tumor promotion [46], and continues to appear in screens for genes that are essential for regulating invasion and migration [47]. Following integrin stimulation, 12-LOX distinctly colocalized with the β4 subunit, predominantly at the edge of cells or at cell-cell junctions. 12-LOX was found localized to cytosolic, perinuclear, and cell surface sites, and the β4 immunofluorescence staining pattern was consistent with its known localization to hemidesmosomes on the ventral surface or the trailing edge of adherent A431 cells [48]. This represents the first identified protein that directly associates with 12-LOX to perhaps scaffold it with additional regulatory proteins residing at the cell surface. The biochemical and imaging data in combination with the 12-LOX knockdown studies provide significant evidence for a physical association between 12-LOX and the cytoplasmic domain of β4 that is functionally relevant for enzymatic activity as demonstrated in the LC-MS and RP-HPLC data, where 12(S)-HETE biosynthesis was increased following specific stimulation of β4. Importantly, this association promotes migration in response to EGF as a chemoattractant. As 12(S)-HETE is known to be stable, the decline that was noted after 60 min is likely due to esterification of the product back into the membrane and not due to degradation.

12(S)-HETE induces a plethora of cellular responses in tumor cells, including protection from apoptosis. Inhibition of 12-LOX activity leads to apoptosis in Walker 256 carcinosarcoma cells, whereas overexpression of 12-LOX in the same cell type results in up-regulation of the anti-apoptotic protein Bcl-2 [28], while in MCF-7 breast cancer cells, overexpression leads to increased cellular proliferation in nude mice [35]. Similarly, addition of exogenous 12-LOX substrate, AA, could rescue human gastric cancer cells from apoptosis induced by serum starvation. This rescue could be blocked by 12-LOX inhibitors, but not by cyclooxygenase pathway inhibitors [28]. Previously we showed that antibody ligation of β4 rescued A431 cells from apoptosis induced by plating cells on an inappropriate growth surface, i.e., untreated polystyrene plates [49]. Therefore, as both 12-LOX, through its metabolite 12(S)-HETE, and β4 ligation have demonstrable anti-apoptotic effects [49], we sought to test the relationship between up-regulation of 12-LOX activity and β4 ligation in relation to cell survival. As demonstrated in the results, ligation of β4 increased resistance of A431 cells to apoptosis induced by 12-LOX inhibitor, BMD122, which appears to support this relationship. Nevertheless, this may not hold true in all cell types, and suggests that the role of β4 in apoptosis may be cell-type specific. Given the wide range of cancer promoting properties of 12-LOX and 12(S)-HETE combined with the tumor promoting functions of β4, targeting their interaction in cancer cells may prove therapeutically efficacious [50]. As noted, β4-mediated, EGF-stimulated A431 cell invasion relied on 12-LOX activation, and the 12-LOX specific enzymatic inhibitor, BMD122, reduced this invasion. 12-LOX knockdown by shRNA rendered the cells un-responsive to EGF-stimulated invasion and resistant to the effects of BMD122. Those residues or motifs in the cytoplasmic domain of β4, or conversely in 12-LOX that are critical for interaction remain to be determined and make an attractive therapeutic target.

## Conclusions

In summary, we have demonstrated that our original discovery using a yeast model of the interaction between the cytoplasmic domain of the β4 subunit of the integrin adhesin and the eicosanoid enzyme 12-LOX is valid, specific, and has functional consequences in mammalian cells. With these data we have demonstrated for the first time that these proteins interact both physically and functionally, thus providing a new paradigm for both integrin and eicosanoid biology. Given the existing correlation between 12-LOX and tumor progression and metastasis, the insight from this study provides the foundation for evaluating this novel interaction with the β4 integrin as a target for intervention.

## Materials and methods

### Antibodies and reagents

Antibodies: to human integrin β4 (mAB1964, clone 3E1; mAB clone 450-11A) and β1 (mAB1951, clone P4G11) subunits, Chemicon International, Inc. (Temecula, CA) of Millipore (Billerica, MA), GIBCO BRL (Gaithersburg, MD), or BD Biosciences/Pharmingen; to human 12-LOX, Oxford Biomedical Research (Oxford, MI); to ERK and phosphorylated ERK (T202/Y204), Cell Signaling (Danvers, MA). Alexa_488_ goat anti-rabbit IgG or Alexa_594_ goat anti-mouse IgG were from Molecular Probes/Invitrogen (Eugene, OR). The anti-β4 mAbs 450-11A and 439-9B were provided by Dr. Steve Kennel (Oak Ridge National Laboratory, Oak Ridge, TN).

Human laminin and EGF were from GIBCO BRL or Sigma Aldrich (St. Louis, MO). The 12-LOX-selective inhibitor, BMD122, formerly called BHPP for N-benzyl-N-hydroxy-5-phenylpentanamide [45], was a generous gift from Biomide Corp. (Grosse Pointe Farms, MI). Other 12-LOX inhibitors: Baicalein, Calbiochem (San Diego, CA); cinnamyl-3,4-dihydroxy-α-cyanocinnamate (CDC), Biomol International, LP (Plymouth Meeting, PA). [^3^H]-12-HETE standard and [^14^C]-AA were from NEN Research Products (DuPont Company, Wilmington, DE). ODS-Silica cartridges were from J.T. Baker Inc. (Phillipsburg, NJ). FuGENE 6 Transfection Reagent kit was from Boehringer Mannheim (Santa Cruz, CA).

### Cell culture and treatments

A431 and Chinese hamster ovary (CHO) cells were obtained from the American Type Culture Collection (Manassas, VA), and cultured as recommended. Transfectants were selected and cultured in media with 300 μg/ml Geneticin (G418; Life Technologies, Inc., Grand Island, NY). Prostate cancer cell lines PC-3 12-LOX/3.1 have been described previously [51].

For treatment with β4 mAb (3E1), 5 × 10^6^ A431 cells were grown to sub-confluence in 100 mm Petri dishes and serum-starved overnight prior to use. Cells were washed with PBS (3×) and stimulated with β4 antibody for 5, 15, 30, 60 and 90 min at a concentration of 5 μg/ml in serum-free DMEM media. For experiments where the natural α6β4 ligand was immobilized, the dishes were coated with laminin (10 μg/ml) and cells were subsequently harvested as above. Otherwise, laminin was used at 5 μg/ml in serum-free media.

### Subcellular fractionation

Cells (5×10^6^) were cultured to 80 % confluence [75-cm^2^ flasks; 37 °C; 5 % CO_2_ in DMEM containing 10 % (v/v) FBS], rinsed (2×) with PBS buffer, and washed (2×) in isotonic buffer (134 mM NaCl; 15 mM Tris–HCl, pH 7.6; 5 mM glucose; 1 mM EDTA; 1 mM EGTA) before suspension in homogenization buffer (25 mM Tris–HCl, pH 7.6; 1 mM EGTA) containing protease inhibitors. Cells were homogenized by sonication (15 sec, 3×, 0 °C) (Vibracell-Microtip) with intervals of 3 min. In some experiments, homogenates were initially centrifuged at 10,000 × g (10 min; 4 °C) and the resultant supernatant was considered cytosolic. The membrane fraction represents the pellet obtained after a one-step centrifugation of the homogenate at 100,000 × g (1 h; 4 °C). The 10,000- and 100,000 × g pellets, respectively, were rinsed once with homogenization buffer and resuspended in protease inhibitor-free homogenization buffer. Samples, standardized by protein concentration, were immediately used for SDS-PAGE.

### Measurement of 12-lipoxygenase activity by LC-MS

12(S)-HETE was measured by liquid chromatography-mass spectrometry as previously described. Cells (8×10^5^) were seeded into six well plates and serum-starved overnight. The following day media was replaced with phenol red-free RPMI media. Cells were then stimulated with 3E1 in the presence of 10 μm AA in 1 % fatty acid-free BSA. AA untreated cells served as a control. As an additional control, AA was incubated in wells without cells to measure spontaneous oxidation of AA into 12(S)-HETE, and this value was subtracted from cell-generated 12(S)-HETE values. The detailed lipid extraction protocol has been described [52]. For measurement of 12(S)-HETE production in parental A431 cells stimulated with 3E1 as a function of time, cells were incubated with 3E1 for the indicated times, washed 1× with serum-free, phenol red-free media, and finally treated with 10 μM AA (in 1 % fatty acid-free BSA) for 15 min. Similarly, media from 12-LOX knock down (KD) cell lines plus control cell lines were collected after 6 h incubation with AA alone, or AA with 3E1 (added together for 6 h). 5 μL of 15-HETE-d8 was added as an internal standard to monitor extraction efficiency. Samples were clarified by centrifugation at 1877 × g for 5 min. Supernatants were subjected to solid phase extraction using Strata-X 33 μm Polymeric Reversed Phase columns (30 mg/1 mL; Phenomenex, Torrance, CA), followed by elution of lipid extracts with methanol, evaporation under a stream of nitrogen, and reconstitution in 50 μL LC-MS grade methanol. Ammonium acetate (50 μL, 35 mM) was added before LC-MS analysis. Samples were analyzed as biological triplicates.

### Immunoprecipitation

Cells were lysed in cold buffer (1 % Triton X-100; 150 mM NaCl; 10 mM Tris, pH 7.4; 1 mM EDTA; 1 mM EGTA, pH 8.0; 0.2 mM sodium ortho-vanadate; 0.2 mM PMSF; 0.01 % aprotinin; 5 μg/ml leupeptin; 0.5 % NP-40), and subsequently clarified (10,000 × g; 10 min). Supernatants were immunoprecipitated with 4–6 μl of antibody against human 12-LOX, anti-β1, or the anti-β4 subunit for 2 h, followed by 40 μl Sepharose 4B-conjugated protein G at 4 °C overnight. Immune complexes were washed (3×) in lysis buffer, and used for SDS-PAGE. Whole cell lysates were used for input controls.

### Western blotting

Performed as per standard techniques with horseradish peroxidase-conjugated secondary anti-IgG diluted 1:4500, and enhanced chemiluminescence (ECL) (both: Amersham, Arlington Heights, IL) for detection.

### Expression constructs and transfection

Dr. Filippo Giancotti (Memorial Sloan-Kettering Cancer Center, NY) kindly provided expression constructs encoding wild-type or mutant, truncated human β4 subunits. These were engineered in the eukaryotic expression vector pRC-CMV (Invitrogen Corp., San Diego, CA) as described [53]; pRC-CMV-β4 (full-length β4 subunit cDNA); pCMV-β4 Δ854-1752 (tail-less, truncated β4 lacking the cytoplasmic domain); and pCMV-β4 Δ70-660 (headless, truncated β4, extracellular sequences replaced by a *c-myc* epitope tag). The full-length cDNA encoding human 12-LOX was subcloned into the EcoRI/XbaI sites of pcDNA3.1 (Invitrogen) from pCMV-12-LOX, gifted from Dr. Colin D. Funk (Queen’s University, Kingston, Ontario, CA). Expression vectors of full-length β4 and 12-LOX used neomycin as a selection marker. As deletion constructs of β4 contained no selective marker, they were cotransfected with neomycin encoding vector, pcDNA3.1. Cells grown in 6-well plates were transfected with 3–12 μg of pcDNA3.1, pCMV-β4, pcDNA-12-LOX, pCMV-β4 tail-less or pCMV-β4 headless using the FuGENE 6 Transfection Reagent following the manufacturer’s protocol. Neomycin-resistant cells were selected in 300 μg/ml geneticin. Knockdown of gene expression by shRNA was performed using Lentiviral pGIPZ constructs targeted to unique regions of the 12-LOX gene, which were purchased from Open Biosystems (Rockford, IL): V2LHS_112083 (#1), V2LHS_112086 (#2), V2LHS_112087 (#3), V3LHS_335849 (#5), V3LHS_335846 (#6), RHS4346 (#8). A431 stable transfections were achieved with 2 μg plasmid DNA, using Lipofectamine LTX (Invitrogen), followed 48 h later by selection in DMEM containing 1 ug/mL puromycin for 3 weeks (Invitrogen, Grand Island, NY). Authorization to use lenti-based vectors for transfection was granted by the WSU Institutional Biosafety Committee as protocol IBC 02-51-11.

### DNA fragmentation assay

Cells (2.5×10^6^) were grown to sub-confluence in 10 cm tissue culture dishes and serum-starved (18 h) prior to use. Cells were washed with PBS (3×), treated with varying BMD122 concentrations for 24 h, and subsequently stimulated with 3E1 antibody for 5, 15, 30, 60 and 90 min. For DNA isolation, cells from each time point were harvested and lysed with lysis buffer (200 μl) for 5 min, clarified at 500 × g (5 min), and the resulting pellet was re-extracted using 200 μl lysis buffer (2 min) and re-clarified. Supernatants were pooled and treated with SDS (1 %) and DNase-free RNase (5 mg/ml) (Ambion, Austin, TX) for 2 h (56 °C), followed by proteinase K (2.5 mg/ml) (Ambion, Austin, TX) treatment for 2 h (37 °C). Finally, samples were extracted (1x) with alkaline phenol/chloroform/isoamyl alcohol (25:24:1) and DNA was precipitated with 0.3 M sodium acetate (pH 5.2) and ethanol. DNA laddering was assayed from equal numbers of cells, or 20 μg resolved on a 1.2 % agarose gel followed by ethidium bromide staining.

### Migration assays

Modified Boyden chambers (Becton Dickinson, Bedford, MA) were coated with human laminin (5 μg/ml; 2 h; 25 °C) on the upper and lower surfaces, and seeded with A431 cells (5x10^5^/ml) in DMEM-0.1 % BSA. Antibodies to β4 integrin were preincubated with aliquots of cells for 20 min prior to seeding. EGF (1 ng/ml) was added to the lower chamber as a chemoattractant. The final concentration of 12-LOX pharmacological inhibitors added to the lower chambers was: 10 μM CDC or baicalein, or 20 μM BMD122. All conditions were tested in triplicate. After 3 h, inserts were fixed in a Quick-Fix solution, double-stained with hematoxylin and eosin (HE), and mounted for observation and counting. The number of migrated cells (12 fields × 100) was counted in a double-blind manner. Alternately, inserts with 8 μm pores (BD Falcon; Franklin Lakes, NJ) were coated with 100 ul of phenol red-free, basement membrane and matrix growth factor-reduced Matrigel (BD Bioscience, Bedford, MA) (250 ug/ml; 1 h; 37 °C; excess liquid removed). Inserts were seeded from confluent, serum-starved (overnight) A431 cells (5 × 10^5^) in 0.5 ml serum-free media. Where noted, cells were pre-treated with 25 μM BMD122 for 1 h prior to 30 min treatments with the following: 3E1 antibody (3 ug/well) or the natural ligand, laminin (10 μg/ml). The lower chamber contained serum-free medium with EGF (2 ng/ml), and complete media with or without serum served as positive and negative controls, respectively. After 24 h, transmigrant cells on the underside of the insert were stained with Azure A&B/Eosin Y using the Diff Quick Stain Kit (IMEB, Inc., San Marcos, CA) and washed twice with distilled water. After removing residual, non-migrated cells, membranes were cut from the inserts, dissolved in 10 % acetic acid and assayed for dye content at an absorbance of OD_570_. Results are the mean of three samples.

### Real-time PCR

Isolated RNA (2 μg) (NucleoSpin RNAII kit; Macherey-Nagel, Bethlehem, PA) was reverse-transcribed (High Capacity Reverse Transcription Kit; Applied Biosystems, Foster City, CA) for real-time PCR (Taqman Gene Expression Master Mix, ALOX12 (HS00167524) and GAPDH primers; Applied Biosystems, Foster City, CA). All sample reactions were run in triplicate on the AB 7500 Fast Real Time PCR System. Relative expression of 12-LOX was quantified by the Ct value measured against the internal standard GAPDH using the 7500 Fast System SDS Software v1.4.0 (Applied Biosystems).

## Additional files

Additional file 1:
**Interaction of 12-LOX with β4 by immunoprecipitation.** The density of the 12-LOX band in transfectants was greater (S1*a*, *right panels*) than the comparable band in the non-transfected cells (Fig. S1*a*, *left panels*). Additional file 2:
**12-LOX colocalization with β4 by laser confocal immunofluorescence imaging.** Subconfluent, serum-starved A431 cells were treated with 5 mg/ml laminin or 3E1 for two hours, or non-specific mouse IgG for one hour. After fixation, cells were labeled sequentially first with P-12 LOX antibody and its respective secondary antibody followed by the anti-b4 antibody and its secondary antibody. Primary and secondary antibodies were used at 1:100 and 1:500, respectively. (Fig. S2*a-d*). Overlapping areas of staining, which appear in yellow in the superimposed confocal images in laminin and 3E1 stimulated cells (Fig. S2*a,b*) were found around the nuclear membrane, at cell-cell junctions and at the cell periphery. In unstimulated controls, or cells treated with mouse preimmune serum, virtually no positive staining was observed for 12-LOX with b4 (Fig. S2*c,d*). The surface staining in green in the IgG treated cells (Fig. S2*d*) either represents a non-specific interaction of the secondary antibody with the IgG used to stimulate the cells, or may represent a novel redistribution of 12-LOX by a component of the pre-immune serum. As controls, 3E1-stimulated cells were stained with secondary antibodies alone (Fig. S2*e*). While anti mouse antibody detected 3E1, used to stimulate the cells, there was limited costaining with the secondary antibodies alone. However, this was rare in the observed fields, and the distribution is different from that seen in the laminin and 3E1-stimulated cells (Fig. S2*a,b, f*; S3-7=.*avi animated Z-stacks*). Mowiol-preserved samples were observed with a Leica TCS SP5 laser scanning confocal microscope. Additional file 3:
**Colocalization of 12-LOX and ITGB4.** Animated selection of confocal microscope Z-stack series corresponding to data in Additional file 2. Additional file 4:
**Colocalization of 12-LOX and ITGB4.** Animated selection of confocal microscope Z-stack series corresponding to data in Additional file 2. Additional file 5:
**Colocalization of 12-LOX and ITGB4.** Animated selection of confocal microscope Z-stack series corresponding to data in Additional file 2. Additional file 6:
**Colocalization of 12-LOX and ITGB4.** Animated selection of confocal microscope Z-stack series corresponding to data in Additional file 2. Additional file 7:
**Colocalization of 12-LOX and ITGB4.** Animated selection of confocal microscope Z-stack series corresponding to data in Additional file 2. Additional file 8:
**Measurement of 12-lipoxygenase activity by RP-HPLC.** (A) 12(S)-HETE peak eluted with corresponding authentic compound. (B) The data shown are the mean value (±SEM) from three experiments as represented in (A), error bars indicate SEM. 12(S)-HETE production was measured using reverse-phase high-performance liquid chromatography (RP-HPLC). At designated time points, 3E1-treated A431 cells (described earlier) were harvested into homogenization buffer and sonicated (10 sec; 2×; 0 °C). Samples were clarified by centrifugation (10,000 × g; 10 min) and supernatants were immediately separated into membrane and soluble fractions by centrifugation (100,000 × g; 4 °C; 1 h). Each fraction (100,000 x g supernatants and resuspended 100,000 x g pellet) was incubated with exogenous ^14^C-AA (10 μM; 37 °C; 15 min). The incubation was terminated by acidification of the suspension to pH 3.5 with 1 N HCl. Samples were centrifuged (2000 × g) and supernatants (cell lipids) were extracted by the method of Benedetto and Lands [54]. Briefly, acidified samples were applied to ODS-Silica cartridges, followed by elution of lipid extracts with freshly redistilled ethyl acetate. These were evaporated under a stream of nitrogen and reconstituted in acetonitrile/acetic acid (1000:1) for HPLC analysis using chromatography conditions based on methods of Powell and Liu. Reverse-phase HPLC was performed using a Beckman Ultrasphere C18-ODS column (4.6 × 250 mm; 5 μm) (Beckman, Fullerton, CA) with a Vista 5500 pump system (Varian, Palo Alto, CA). Lipoxygenase metabolites of AA were resolved in an isocratic solvent system of acetonitrile/water/acetic acid [54:46:0.05] at 1.5 ml/min. Column effluent was continuously monitored with a Varian 2550 UV/Vis spectrophotometer (Varian) set at 236 nm and a radioisotope flow detector (β-RAM, IN/US, Fairfield, NJ). The lipoxygenase metabolites were identified based on the retention time of the authentic compounds. [^3^H]-12(S)-HETE was used to confirm the identity of the peak in the sample. Additional file 9:
**Detection of nucleosomes in the cytoplasm of cells treated with BMD122.** A431 cells were exposed for 48 h to different concentrations of BMD122. After cell lysis and centrifugation, the cytoplasmic fractions were prediluted 1:10 with incubation buffer and tested for nucleosomes by ELISA. Substrate reaction time: 15 min. Additional file 10:
**BMD122 effects on A431 cell survival by Trypan blue exclusion assay.** The maximum cell killing (i.e., the lowest cell survival) was noticed at 100 μM BMD122. The treatment was 48 h, and the results are expressed as % cell survival compared to ethanol control (i.e., 0 mM BMD122). Each condition was run in triplicate, and the results were derived from the mean +/− SE of three independent experiments.

## Abbreviations

LOX: Lipoxygenase
HETE: Hydroxyeicosatetraenoic acid
BHPP: Benzyl-N-hydroxy-5-phenylpentanamide
BMD122: Biomide compound 122
CDC: Cinnamyl-3,4-dihydroxy-α-cyanocinnamate
LN: Laminin
TBS: Tris buffered saline
ECL: Enhanced chemiluminescence
