# Supplementary figures and images for: Convergence of eicosanoid and integrin biology: 12-lipoxygenase seeks a partner
Source: Mol Cancer. 2015 Jun 3;14:111. doi: 10.1186/s12943-015-0382-5 (PMC4453211; doi:10.1186/s12943-015-0382-5)

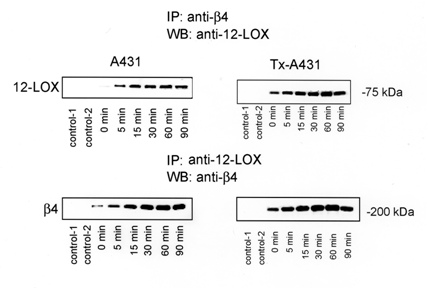

Supplement: Additional file 1: — Interaction of 12-LOX with β4 by immunoprecipitation. The density of the 12-LOX band in transfectants was greater (S1a, right panels) than the comparable band in the non-transfected cells (Fig. S1a, left panels). [file 12943_2015_382_MOESM1_ESM.tiff]

## Slide 1
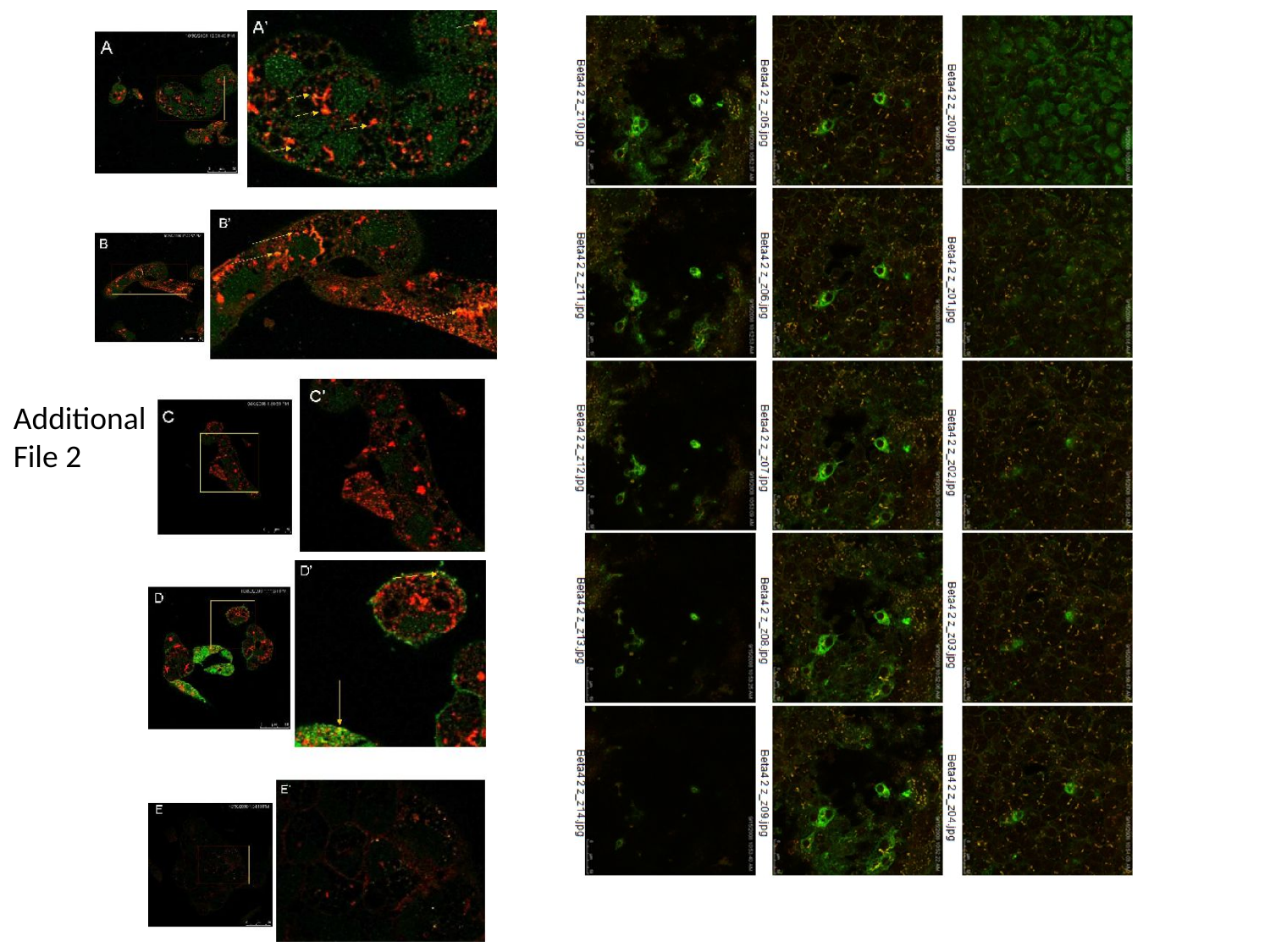

Additional
File 2

Supplement: Additional file 2: — 12-LOX colocalization with β4 by laser confocal immunofluorescence imaging. Subconfluent, serum-starved A431 cells were treated with 5 mg/ml laminin or 3E1 for two hours, or non-specific mouse IgG for one hour. After fixation, cells were labeled sequentially first with P-12 LOX antibody and its respective secondary antibody followed by the anti-b4 antibody and its secondary antibody. Primary and secondary antibodies were used at 1:100 and 1:500, respectively. (Fig. S2a-d). Overlapping areas of staining, which appear in yellow in the superimposed confocal images in laminin and 3E1 stimulated cells (Fig. S2a,b) were found around the nuclear membrane, at cell-cell junctions and at the cell periphery. In unstimulated controls, or cells treated with mouse preimmune serum, virtually no positive staining was observed for 12-LOX with b4 (Fig. S2c,d). The surface staining in green in the IgG treated cells (Fig. S2d) either represents a non-specific interaction of the secondary antibody with the IgG used to stimulate the cells, or may represent a novel redistribution of 12-LOX by a component of the pre-immune serum. As controls, 3E1-stimulated cells were stained with secondary antibodies alone (Fig. S2e). While anti mouse antibody detected 3E1, used to stimulate the cells, there was limited costaining with the secondary antibodies alone. However, this was rare in the observed fields, and the distribution is different from that seen in the laminin and 3E1-stimulated cells (Fig. S2a,b, f; S3-7=.avi animated Z-stacks). Mowiol-preserved samples were observed with a Leica TCS SP5 laser scanning confocal microscope. [file 12943_2015_382_MOESM2_ESM.pptx]

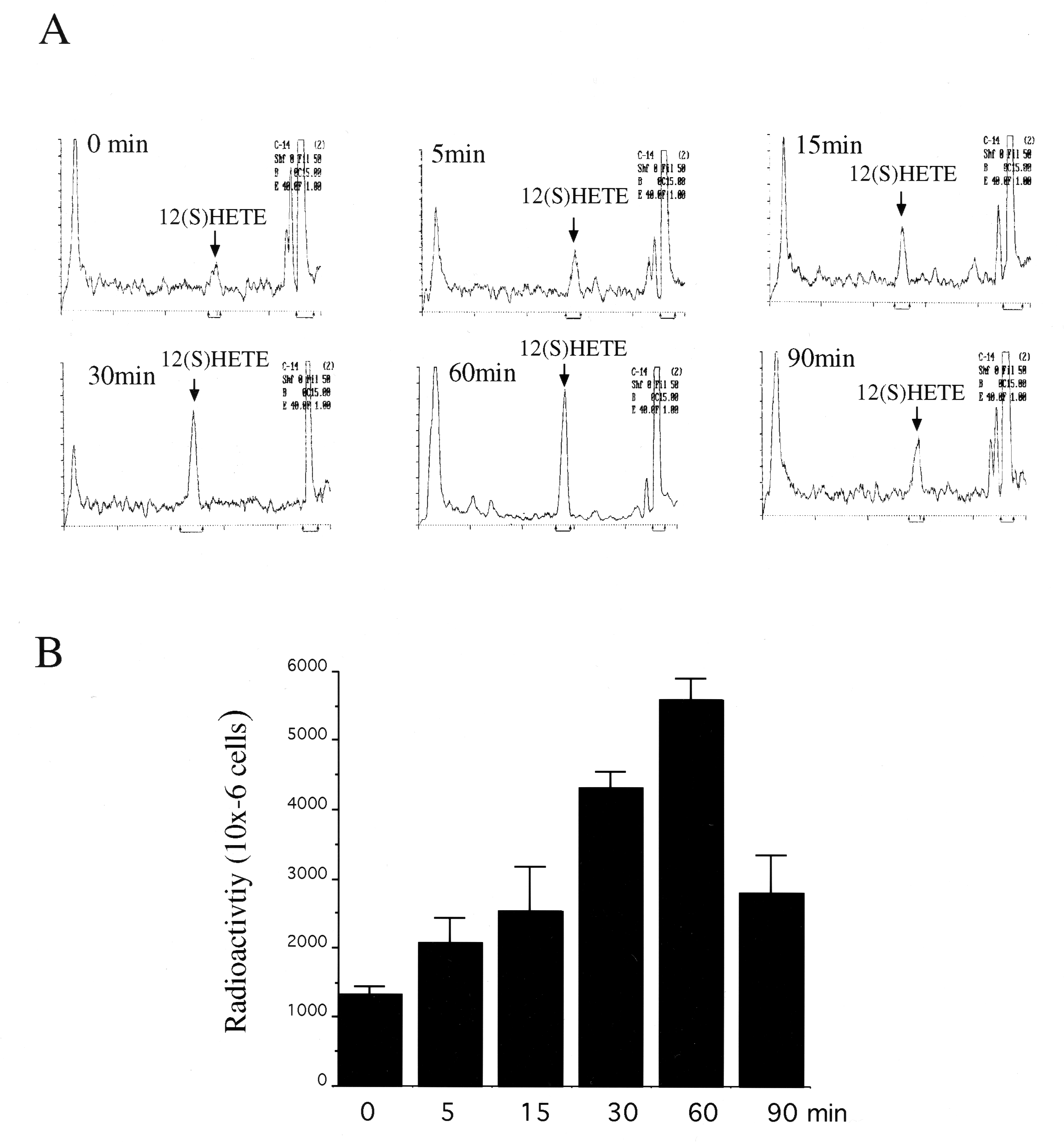

Supplement: Additional file 8: — Measurement of 12-lipoxygenase activity by RP-HPLC. (A) 12(S)-HETE peak eluted with corresponding authentic compound. (B) The data shown are the mean value (±SEM) from three experiments as represented in (A), error bars indicate SEM. 12(S)-HETE production was measured using reverse-phase high-performance liquid chromatography (RP-HPLC). At designated time points, 3E1-treated A431 cells (described earlier) were harvested into homogenization buffer and sonicated (10 sec; 2×; 0 °C). Samples were clarified by centrifugation (10,000 × g; 10 min) and supernatants were immediately separated into membrane and soluble fractions by centrifugation (100,000 × g; 4 °C; 1 h). Each fraction (100,000 x g supernatants and resuspended 100,000 x g pellet) was incubated with exogenous 14C-AA (10 μM; 37 °C; 15 min). The incubation was terminated by acidification of the suspension to pH 3.5 with 1 N HCl. Samples were centrifuged (2000 × g) and supernatants (cell lipids) were extracted by the method of Benedetto and Lands [54]. Briefly, acidified samples were applied to ODS-Silica cartridges, followed by elution of lipid extracts with freshly redistilled ethyl acetate. These were evaporated under a stream of nitrogen and reconstituted in acetonitrile/acetic acid (1000:1) for HPLC analysis using chromatography conditions based on methods of Powell and Liu. Reverse-phase HPLC was performed using a Beckman Ultrasphere C18-ODS column (4.6 × 250 mm; 5 μm) (Beckman, Fullerton, CA) with a Vista 5500 pump system (Varian, Palo Alto, CA). Lipoxygenase metabolites of AA were resolved in an isocratic solvent system of acetonitrile/water/acetic acid [54:46:0.05] at 1.5 ml/min. Column effluent was continuously monitored with a Varian 2550 UV/Vis spectrophotometer (Varian) set at 236 nm and a radioisotope flow detector (β-RAM, IN/US, Fairfield, NJ). The lipoxygenase metabolites were identified based on the retention time of the authentic compounds. [3H]-12(S)-HETE was used to confirm the identity of the [file 12943_2015_382_MOESM8_ESM.tiff]

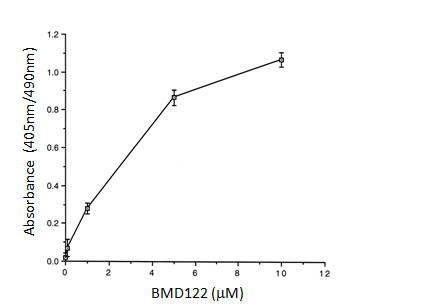

Supplement: Additional file 9: — Detection of nucleosomes in the cytoplasm of cells treated with BMD122. A431 cells were exposed for 48 h to different concentrations of BMD122. After cell lysis and centrifugation, the cytoplasmic fractions were prediluted 1:10 with incubation buffer and tested for nucleosomes by ELISA. Substrate reaction time: 15 min. [file 12943_2015_382_MOESM9_ESM.tiff]

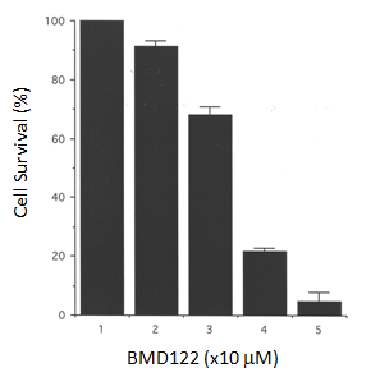

Supplement: Additional file 10: — BMD122 effects on A431 cell survival by Trypan blue exclusion assay. The maximum cell killing (i.e., the lowest cell survival) was noticed at 100 μM BMD122. The treatment was 48 h, and the results are expressed as % cell survival compared to ethanol control (i.e., 0 mM BMD122). Each condition was run in triplicate, and the results were derived from the mean +/− SE of three independent experiments. [file 12943_2015_382_MOESM10_ESM.tiff]
